# Accelerated 23-h enhanced recovery protocol for colon surgery: the CHASE-study

**DOI:** 10.1038/s41598-022-25022-7

**Published:** 2022-12-01

**Authors:** Thaís T. T. Tweed, Misha A. T. Sier, Imane Daher, Maikel J. A. M. Bakens, Johan Nel, Nicole D. Bouvy, James van Bastelaar, Jan H. M. B. Stoot

**Affiliations:** 1 Department of Gastrointestinal Surgery, Zuyderland Medical Center, Dr. H. van der Hoffplein 1, 6162 BG Sittard-Geleen, The Netherlands; 2 Department of Surgery, Maastricht University Medical Center, P. Debeyelaan 25, 6229 HX Maastricht, The Netherlands

**Keywords:** Surgery, Health policy, Patient education

## Abstract

The introduction of the Enhanced Recovery After Surgery (ERAS) program has radically improved postoperative outcomes in colorectal surgery. Optimization of ERAS program to an accelerated recovery program may further improve these said outcomes. This single-center, prospective study investigated the feasibility and safety of a 23-h accelerated enhanced recovery protocol (ERP) for colorectal cancer patients (ASA I–II) undergoing elective laparoscopic surgery. The 23-h accelerated ERP consisted of adjustments in pre-, peri- and postoperative care; this was called the CHASE-protocol. This group was compared to a retrospective cohort of colorectal cancer patients who received standard ERAS care. Patients were discharged within 23 h after surgery if they met the discharge criteria. Primary outcome was the rate of the successful discharge within 23 h. Successful discharge within the CHASE-cohort was realized in 33 out of the 41 included patients (80.5%). Compared to the retrospective cohort (n = 75), length of stay was significantly shorter in the CHASE-cohort (*p* = 0.000), and the readmission rate was higher (*p* = 0.051). Complication rate was similar, severe complications were observed less frequently in the CHASE-cohort (4.9% vs. 8.0%). Findings from this study support the feasibility and safety of the accelerated 23-h accelerated ERP with the CHASE-protocol in selected patients.

## Introduction

Introduction of the Enhanced Recovery After Surgery (ERAS) protocol has revolutionized perioperative colorectal surgical care^1^. The ERAS protocol consists of evidence-based interventions to reduce perioperative stress, maintain postoperative physiological functioning and accelerate recovery after surgery^1^. Studies have shown that the combination of ERAS protocol and laparoscopic surgery have resulted in a considerable reduction in length of hospital stay (LOS) and morbidity, faster recovery without increasing readmission rate, and cost reduction when compared to traditional care^2–8^. Notwithstanding this significant improvement in modern day surgery, there is an interest in further optimization of the recovery pathway.

Recent studies have demonstrated that an accelerated enhanced recovery protocol (ERP), with discharge on the day of surgery to three days after surgery, is feasible for specific patients without compromising patients’ safety or increasing postoperative risks^9–16^. These results were obtained from studies that have explored ERPs with a modified preoperative, anaesthetic and postoperative protocol, or modified postoperative care with home visits or monitoring using a smartphone application^12^.

Although the evidence is limited, optimizing the ERP appears to be feasible. To date, uncertainty remains regarding the optimal format of an accelerated ERP. There is a certain ambiguity on which patients are suitable for the accelerated ERP and what benefits this protocol offers surgical patients. Moreover, the advantageous impact on increasing healthcare costs remains speculative^6^.

The aim of this study is to determine feasibility and safety of the CHASE protocol, a 23-h admission enhanced recovery protocol, for patients undergoing colon surgery.

## Methods

### Study design

This prospective, single-center study was conducted in a large Dutch teaching hospital (Zuyderland Medical Center, the Netherlands). Patients diagnosed with malignant colon disease were assessed for eligibility and enrolled in the surgical outpatient clinic between June 2020 and August 2021. Patient data were collected at baseline, perioperatively and at 30-days follow-up. Patients operated for colon cancer in 2018, who met the inclusion criteria, served as a control group.

### Study population

Patients diagnosed with (non-complicated) colon cancer upon colonoscopy were screened for eligibility and included if they met the following inclusion criteria: aged ≥ 18 years ≤ 80, American Society of Anesthesiologists (ASA) classification I-II, BMI ≤ 35 kg/m2, readily available ambulant care (provided by an adult family member) for the first 24 h after discharge and adequately reachable by phone. Furthermore, patients had to be scheduled for an elective colon resection (right or left hemicolectomy, transverse colon resection, sigmoid resection) with primary anastomosis performed intracorporeally.

Exclusion criteria were ASA classification ≥ III, limited mobility and/or in need of assisted mobilizing, a history of an active pulmonary infection or any other active infection or uncontrolled medical disease, contra-indication for oral Non-Steroidal Anti-Inflammatory Drugs (NSAID) or spinal anesthesia, parenteral nutrition required prior to surgery, mental or physical limitations in activities of daily living, rectal resection or creation of an ostomy, and perioperative complications.

Ethical approval was obtained from the local Medical Research Ethics Committee (MREC) Zuyderland (NL71804.096.19). This study was conducted according to the ethical standards of the Helsinki Declaration of 1975^17^. All patients provided written informed consent prior to inclusion.

### Baseline assessment

After inclusion, all patients were screened by the anesthesiologist. This screening consisted of a complete cardiopulmonary physical examination, electrocardiogram on indication and routine laboratory workup. Patients were excluded from the study if any contraindication for participation was present.

In addition, all patients underwent routine preoperative work-up, which consisted of a standard consultation with the clinical dietician who would screen for malnutrition with the Short Nutritional Assessment Questionnaire (SNAQ) and a consultation with the nurse practitioner for information and education about the CHASE study and protocol^18^. This included patient expectation management and screening of patient frailty with the Groningen Frailty Indicator (GFI)^19^.

### The CHASE-protocol

The 23-h ERP of Levy et al.^9^ was minimally adjusted and introduced for this study. This protocol comprised of modifications in the pre-, peri- and postoperative care. All steps of the protocol are described in Table 1. Initially, patients received a combination of spinal anesthesia with bupivacaine-glucose and morphine intrathecally prior to general anesthesia. After the first 9 included patients, this was switched to intrathecal Prilocaine in combination with total intravenous anesthesia (TIVA), because of an increase in the incidence of nausea and urinary retention postoperatively. The remaining included patients received spinal anesthesia with 60-80 mg Prilocaine (weight based).

**Table 1:** CHASE Enhanced Recovery Protocol.

**Preoperative**	Dedicated preoperative counseling, which entailed i.e. expectation management by the anesthesiologist; Baseline assessment with physical examination, electrocardiogram on indication and standard laboratory work-up; Nutritional screening by dietician, which included the Short Nutritional Assessment questionnaire; Fasting 6 h prior to surgery for solid food and 2 h prior to surgery for liquids Oral carbohydrate loading for non-diabetic patients at least 2 h prior to surgery; Bowel preparation with bisacodyl (2 tablets of 5 mg the night before surgery and 1 tablet the morning of surgery) for patients scheduled for left-sided colon surgery Patients were admitted at 07:00 AM to the surgical ward; their operation scheduled as second or third operation of the day; Preoperative analgesia with 1000 mg Paracetamol and 600 mg Gabapentin (300 mg if glomerular filtration rate < 60 ml/min or age > 70 years); Ambulation to the operation theatre
**Intraoperative**	Spinal anesthesia (Prilocaine) prior to induction of general anesthesia; The use of short-acting total intravenous anesthesia (propofol, remifentanil and ketamine (analgesic dosage) in combination with the pre-induction spinal anesthesia; Restrictive fluid management with continuous perfusion of Ringer Lactate 3 ml/kg/h; Deep neuromuscular blockade (Rocuronium bromide perfusion) Lung protective ventilation (Total Volume 6–8 ml/kg; minimum FiO2 and optimal PEEP) Adequate temperature regulation with forced air warming and core temperature monitoring; Starting intra-abdominal pressure at 12 mmHg with a gradual decrease to 8 mmHg^29,30^ Minimally invasive surgery with intracorporal anastomosis^31–37^ Extraction of specimen through a suprapubic Pfannenstiel incision, no additional mini-laparotomy performed^38^
**Postoperative—Directly**	Postoperative pain management consisted of Paracetamol(4 × 1000 mg), Meloxicam (1 × 7.5 mg daily for 3 days). If indicated, 5–20 mg Oxynorm (per os) or opioids in the form of Piritramide (intravenously) were given; Intake and gastro-intestinal motility were stimulated by offering an ice lolly on the recovery ward; On the surgical ward, intake and mobilization were actively stimulated; If an urinary catheter was placed; this was removed before 10:00 PM on the surgical ward;
**Postoperative day (POD) 1**	Routine physical examination by the ward physician; Evaluation of recovery and readiness for discharge. Patients were considered ‘functionally recovered’ and safe for discharge if they met the following criteria: Adequate analgesia with oral analgesics, VAS < 4; No symptoms of nausea or vomiting; Presence of flatus or the passing of stool; Oral intake possible; Spontaneous micturition; Ability to mobilize independently; Absence of abnormal vital signs (e.g. fever, tachycardia, hypotension, dyspnea or somnolence) Expectation management regarding postoperative recovery and provision of an information booklet about postoperative recovery
**After discharge**	Telephonic aftercare was conducted by the nurse on the evening of discharge (POD 1) and by the nurse practitioner on postoperative day 4 to evaluate recovery and assess the presence of abnormal vital signs; Appointment in outpatient clinic one week after discharge to receive the pathology results and consequent treatment plan Evaluation of patients’ experience with the CHASE protocol (see Supplementary form S1.1)

### Outcomes

The primary study outcome was the success-rate of the 23-h accelerated enhanced recovery protocol. The success was defined as a minimum of three quarters (75%) of patients who could be discharged home on postoperative day 1.

Secondary outcome measures were readmission, postoperative complications and mortality rate ≤ 30 days after surgery. In addition, safety was measured by means of incidence of severe postoperative complications defined as Clavien Dindo (CD) IIIb and above. The patient satisfaction was assessed in an evaluation form (see supplementary form S1.1).

Outcomes of the CHASE population were compared to a retrospective cohort of patients operated in 2018.

### Statistical analysis

To study the success and safety of this ERP, the aim was to include thirty patients. By adjusting the medication for the spinal anesthesia, the study population was expanded to 40 patients. Normality was tested using the Shapiro–Wilk test. Normally distributed continuous variables were presented as means with the standard deviation (SD) and non-normally distributed variables as medians with the Inter Quartile Range (IQR). Depending on the normality of data, differences between the two groups were examined using independent sample T-test or Mann–Whitney-U test for continuous data. The Chi-square test and Fisher exact test were used to analyze differences in binary and nominal data.

Multivariate regression analysis was performed to evaluate the effect of the CHASE protocol on length of hospital stay, complication and readmission rate. Confounding factors were adjusted for in these analyses included the following: age, gender, Charlson Comorbidity Index (CCI)^20–24^. Statistical analyses were performed using Statistical Package for the Social Sciences for Windows (version 25.0; IBM, SPSS Inc., Chicago, IL, USA). Statistical significance was set at *p* < 0.05.

## Results

**Figure 1:**
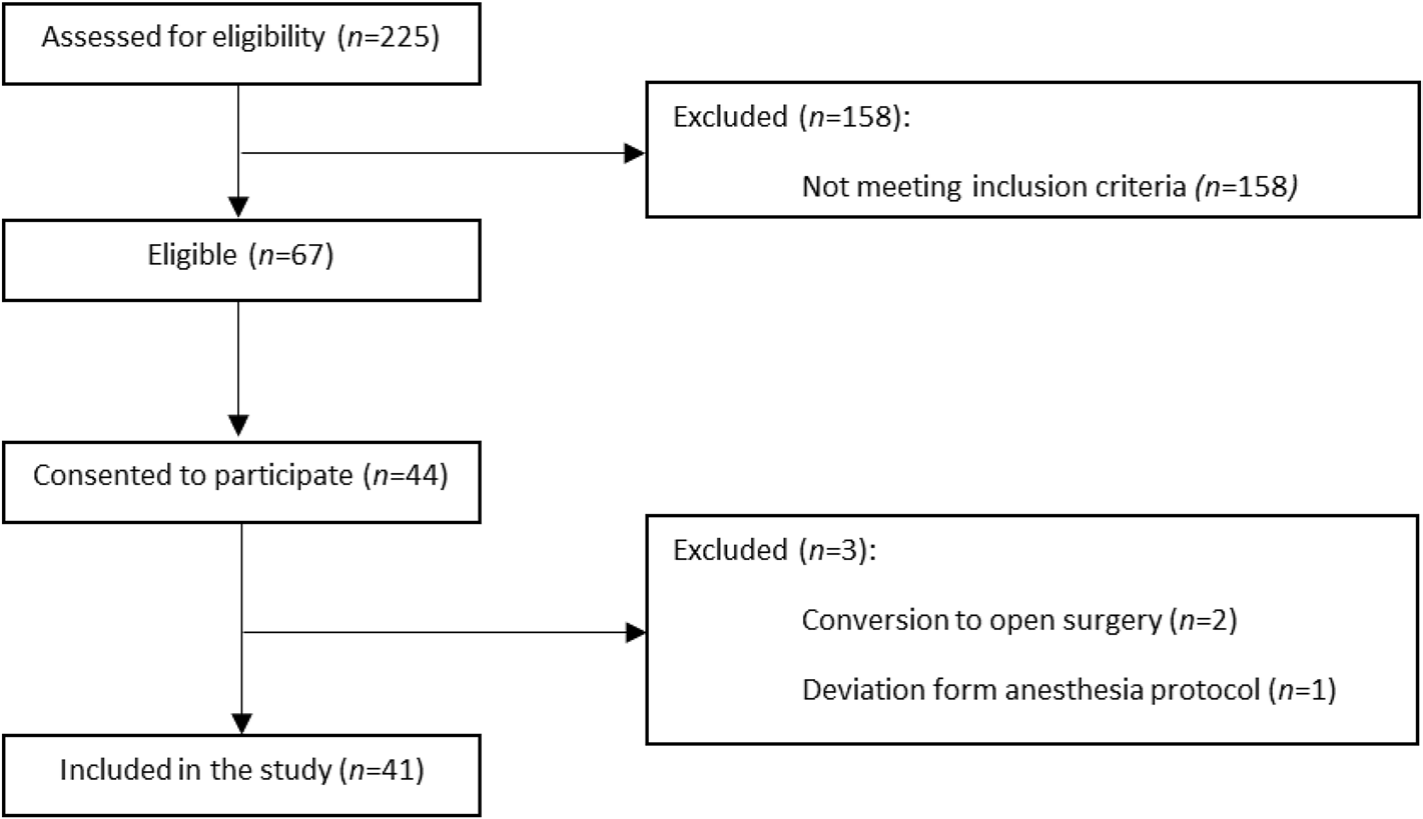
Consort diagram. Flowchart CHASE cohort.

**Figure 2:**
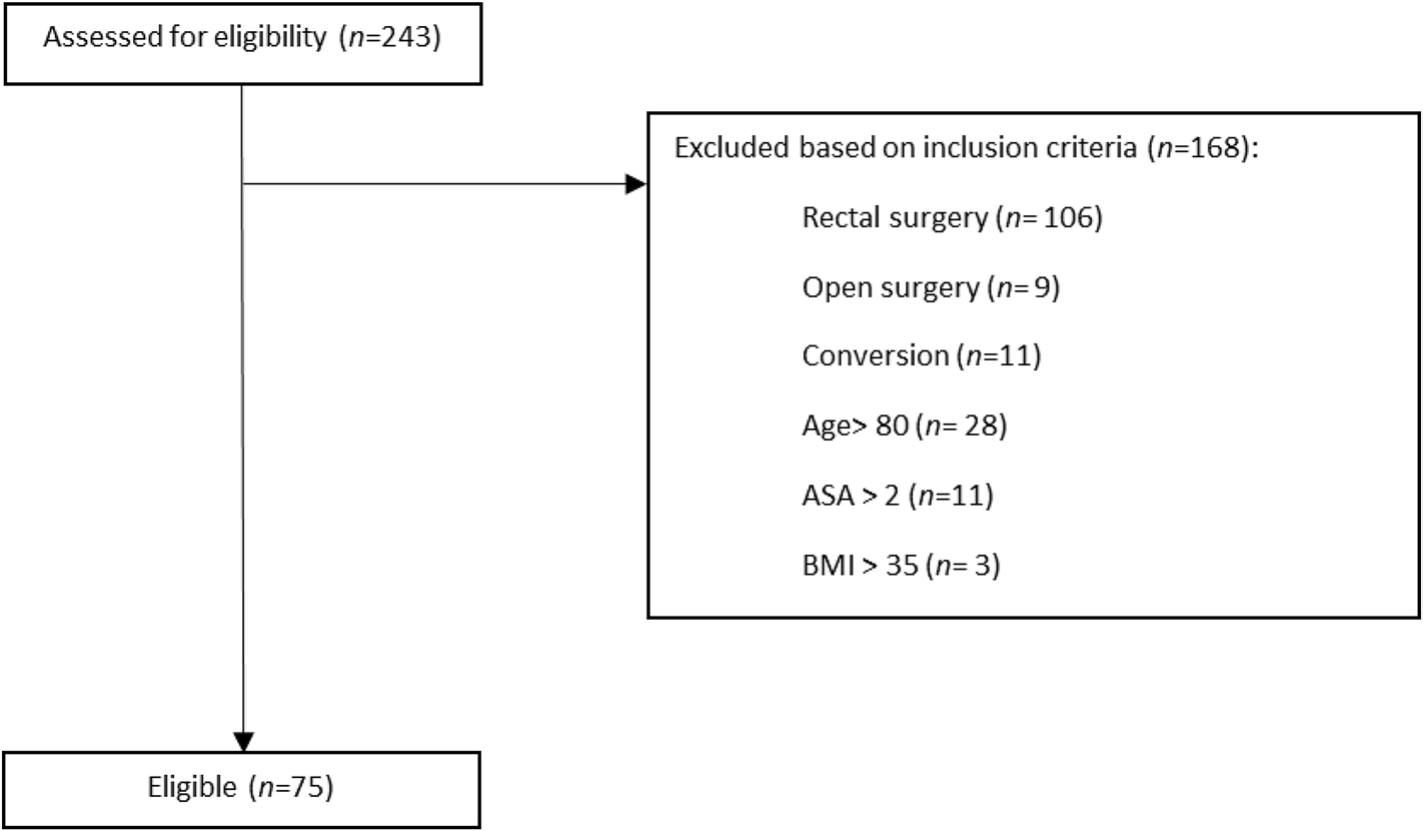
Consort diagram. Flowchart retrospective cohort.

Between the 1st of June 2020 and the 13th of August 2021, a total of 225 patients were assessed for eligibility for participation. Sixty-seven patients met the inclusion criteria of which 44 consented to participate. Three patients were excluded; two patients due to conversion to open surgery and one patient due to deviation from the anesthesia protocol. A flowchart for the CHASE cohort is presented in Fig. 1. Of the historical cohort, patients operated in 2018, 75 patients met the inclusion criteria for the CHASE study. Figure 2 shows the flowchart for this retrospective cohort.

### Patient characteristics

Demographic characteristics of the study patients and retrospective cohort are shown in Table 2. Patients were significantly younger (median 67.0 versus median 72.6 years), more likely to be male (65.9% versus 44.0%), and had a lower Charlson Comorbidity Index score (mean 4.22 versus 5.03) in the CHASE cohort compared to the retrospective cohort. Other demographic characteristics were mostly similar. No differences in surgical procedures were observed.

**Table 2:** Patient characteristics; median (interquartile range).

	CHASE cohort (n = 41)	Retrospective cohort (n = 75)	*p*-value
**Age (years)**	67.0 (57.0–73.0)	72.6 (63.6–75.7)	0.007
**Sex (male)**	27 (65.9%)	33 (44.0%)	0.024
**BMI**^**1**^ **(kg/m**^**2**^**)**	26.4 (23.0–29.3)	25.6 (23.6–29.0)	0.797
**Comorbidity; n (%)**	24 (58.5%)	53 (70.7%)	0.186
**History of abdominal surgery**	14 (34.1%)	24 (32.0%)	0.814
**Cardiovascular disease**	10 (24.4%)	41 (54.7%)	0.002
**Pulmonary disease**	4 (9.8%)	9 (12.0%)	1.000
**ASA**^**2**^** score; n (%)**			0.156
I	1 (2.4%)	8 (10.7%)	
II	40 (97.6%)	67 (89.3%)	
**CCI**^**3**^; **mean (SD)**	4.22 (1.24)	5.03 (1.48)	0.004
**Surgical procedure; n (%)**			
Left hemicolectomy	3 (7.3%)	6 (8.0%)	
Right hemicolectomy	19 (46.3%)	28 (37.3%)	
High anterior resection	18 (43.9%)	37 (49.3%)	
Transverse colectomy	1 (2.4%)	1 (1.3%)	
Total colectomy	0	1 (1.3%)	
Subtotal colectomy	0	2 (2.6%)	

^1^*BMI* body mass index; ^2^*ASA* American Society of Anesthesiologists physical status classification system; ^3^*CCI* Charlson Comorbidity Index score; SD: standard deviation.

### Primary outcome

#### Length of hospital stay

Of the 41 patients included in the “CHASE” ERP, 33 (80.5%) patients were discharged on postoperative day 1 of the initial admission. All patients were discharged within 3 days after surgery. Reasons for prolonged admission were rectal blood loss (n = 3), bladder retention (n = 2), pain (n = 2) and nausea (n = 1) (see Table 3 and Supplementary Table S1.2). Compared to the retrospective cohort, length of hospital stay was significantly shorter in the CHASE cohort (*p* = 0.000), see Table 3.

**Table 3:** Hospital and surgical data; primary and secondary outcomes.

	CHASE population (n = 41)	Retrospective cohort (n = 75)	* p*-value
Primary length of hospital stay*			
1 day	33 (80.5%)	0	0.000
2 days	6 (14.6%)	9 (12.0%)	
3 days	2 (4.9%)	38 (50.7%)	
> 3 days	0	28 (37.3%)	
**Re-admission < 30 days**	7 (17.1%)	4 (5.3%)	0.051
**Readmission of patients discharged ≤ 23 h (n = 33)**	6 (18.2%)	NA	
Length of stay readmission*	5 (IQR 2–10)	6 (IQR 4–7)	0.870
**Complication; n (%)**	13 (31.7%)	20 (26.7%)	0.565
**Complication among patients discharged ≤ 23 h (n = 33)**	9 (27.3%)	NA	
**Clavien Dindo grade**			0.867
0	28 (68.3%)	56 (74.7%)	
I–IIIa	11 (26.8%)	13 (17.3%)	
IIIb–IV	2 (4.9%)	6 (8.0%)	
**Re-intervention within 30 days**	2 (4.9%)	8 (10.0%)	0.491
**30-day mortality; n**	0	0	

*NA* not applicable; *In median days.

### Secondary outcome

#### Readmission

Over the course of this study, seven patients (17.1%) were readmitted. Six of the readmissions occurred among the 33 patients discharged within 23 h after surgery (18.2%). Main reasons for readmission were ileus (n = 2) and rectal blood loss (n = 2). Readmission rate was higher in the CHASE population (17.1% vs. 5.3%, *p-*value 0.051). In the CHASE cohort, all readmissions occurred within 2 days after discharge.

#### Surgical outcomes

Thirteen patients (31.7%) developed postoperative complications within 30 days after surgery: most often gastro-intestinal complications, such as an ileus (see Supplementary Table S1.3). Of the patients discharged within 23 h, nine patients developed a complications (27.3%). Serious adverse events (CD ≥ IIIb) occurred in two patients (4.9%) in the CHASE cohort requiring reoperation; one patient developed an anastomotic leakage and one patient suffered from a trocar hernia. Median length of hospital stay during readmission was 5 days for patients who were readmitted. Reasons for readmission are shown in Supplementary Table S1.4.

In comparison to the retrospective cohort, no difference was observed in complication rate (*p-*value = 0.565), but severe complications (CD≥ IIIb) were observed more frequently in the retrospective cohort. Six patients in the control group suffered from serious adverse events; four patients developed anastomotic leakage, one patient developed small bowel ischemia, and one patient an abscess which required drainage. No patients died within 30 days after surgery in both groups.

Multivariate regression analysis was used to test if the CHASE protocol had a significant effect of the length of stay when correcting for confounders. After correction for the confounding factors (age, sex, CCI) the CHASE protocol demonstrated to significantly reduce length of hospital stay (*p* = 0.000). Readmission rate (*p* = 0.067, odds-ratio 3.673) and complication rate (*p* = 0.652, odds-ratio 1.229) did not statistically differ between groups after correction for confounders.

#### Patient satisfaction

Evaluation forms assessing patients’ satisfaction were returned by 39 patients (95%). The CHASE protocol was positively reviewed by the majority of patients. Eighty-seven percent of patients rated the program as an 8 or higher on a scale of 10.

## Discussion

The results of this study demonstrated feasibility and safety to discharge selected patients within 23 h after colon surgery with help of an accelerated enhanced recovery protocol.

The “CHASE” protocol entailed modifications of the enhanced recovery protocol by the adaptation of three key factors; minimally invasive surgery with intracorporeal anastomosis; optimal fluid management, and optimal analgesia through a combination of spinal-general anesthesia technique (TIVA) with short acting opioids. Results of the CHASE study demonstrated the success of this protocol as 33 patients out of 41 patients were successfully discharged on postoperative day 1. Patients treated according to the CHASE protocol experienced a lower rate of severe complications (CD ≥ IIIb). Overall, length of hospital stay was significantly shorter in the CHASE cohort. Both postoperative complications and readmissions did not statistically differ between groups after correcting for confounders upon multivariate regression analysis.

### Successful and safe application CHASE protocol

Discharge on postoperative day 1 was successful for 80.5% of the patients in the CHASE cohort, effectuating a significant decrease in LOS when compared to the retrospective cohort. The LOS in the Zuyderland Medical Center had already been improved by peri-operative education of expected day of discharge^25^. The high rate of successful discharge on postoperative day 1 is similar to the report of Levy et al. who found that discharge on postoperative day 1 or on day of surgery was feasible for all the ten included patients^9^.

Readmission rate observed in the CHASE population was higher than in the retrospective cohort. Nonetheless, considering the reasons for readmissions the relationship with the CHASE protocol is highly debatable. Importantly, readmission did not affect morbidity as complication rates were similar in both groups and more severe complications occurred in the retrospective cohort. Previously reported readmission rates following accelerated enhanced recovery varied from 0–14%^9,11,12,26^. The higher rate of readmission in the CHASE cohort (17.1%) must be interpreted with caution as the reason for readmission may not be related to the CHASE protocol. Differences with previous studies can be explained by the fact that other studies had a smaller study population^9,26^, a younger patient population^9,11,12,26^ and also included patients with benign pathology^9,11,12^ and patients undergoing stoma closure^12^. Other factors that could have contributed to fewer readmissions involve inclusion of robotic resections^26^, and exclusion of patients with an age > 75 years, a BMI > 28^9^, or significant comorbidities^11,12,26^. Some studies also excluded patients with a history of perforated sigmoid diverticulitis or midline laparotomy^11^, or a history of severe postoperative nausea or vomiting^9,26^. Furthermore, patients in the study of Gignoux et al. received daily ambulatory care by the homecare nurse for the first 10 postoperative days and laboratory analysis was performed on postoperative days 1, 3 and 7. Patients in the study of Lee et al. were requested to fill in an online survey on a daily basis upon postoperative day 7^12^. These interventions potentially reduced the need for readmission. On the other hand these studies did include patients with rectal cancer requiring more complex surgical resections or patients with ASA score of III^11,12,26^.

The protocol for accelerated recovery varied between studies; an important difference was the administration of local anesthesia or an abdominal plane block^11,12^ instead of spinal anesthesia. No information regarding abdominal pressure during surgery was given^9,11,12,26^.

A key concern of accelerated recovery is that patients are exposed to a major risk if they develop life-threatening complications within the early postoperative period while they are at home. Results of this study demonstrated the safety of the CHASE protocol since the complication rate did not differ between both groups and the number of severe complications decreased. The morbidity rate and the low incidence of severe complications is consistent with the literature^11,12,26^.

When comparing the CHASE study to other studies, informing patients about the benefits of reduced hospital stay, adequate pain management and stimulation of intake and mobilization were also frequently implemented in other accelerated recovery pathways^11,12,26^. Studies determining the effects of low intra-abdominal pressure during laparoscopy demonstrated reduction of postoperative pain scores^27,28^. Presumably, all these factors have contributed, to a greater or lesser extent, to the rapid recovery.

### Limitations

The results of this study must be interpreted with awareness of several limitations. First, the design of this study is a prospective single center cohort study. We compared the outcomes with a retrospective cohort from 2018 with an improved ERP by means of peri-operative education of expected day of discharge^25^. Although inclusion criteria were strict, which resulted in a rather homogeneous population, the moment of surgery and admission might have affected outcomes. Moreover, this study involves a small study sample, which could limit the applicability of the results to the entire patient population. In addition, study patients were selected, with less than half of patient eligible for the CHASE protocol. Generalizability to other patient populations may be limited, nonetheless the majority of eligible patients were willing to participate in this study. The proximity and accessibility of referral hospitals in the Netherlands are favorable, which could have aided in the safety aspect of this protocol. Perhaps for other (non) Western countries with longer distances between patient residence and hospital; the CHASE protocol would be less attractive.

The first nine patients included in this study received local anesthetics and spinal anesthesia with Morphine intrathecally. Due to the increase in nausea and urine retention, spinal anesthesia with Prilocaine intrathecally and total intravenous anesthetic substituted the spinal anesthesia with Morphine. This resulted in a decrease in side effects. As this modification during the study might have affected the postoperative outcomes, the study population was extended with ≥ ten patients (from thirty patients to forty-one patients). The CHASE protocol consisted of several modifications to the current ERAS protocol, therefore this study is not able to identify the factor(s) that had the highest impact on the success and safety of the accelerated recovery with the CHASE protocol.

### Strengths

Strengths of this study include the detailed ERP which was strictly adhered to, a well-trained multidisciplinary team which consisted of all involved healthcare providers and which was easy to reach for the patients.

Future studies should focus on assessing the success and safety of this accelerated recovery protocol in a larger study population. Moreover, the applicably of this study protocol to other patient populations, e.g. ASA III or older patients, could be explored. In further investigations, evaluation cost-effectiveness of accelerated recovery is recommended.

## Conclusion

This was the first reported study conducted in the Netherlands to assess the feasibility and safety of a 23-h enhanced recovery protocol for colorectal cancer surgery. This study demonstrated that the CHASE enhanced recovery protocol is successful and safe for selected patients undergoing minimally invasive surgery for colon cancer.

Most patients were discharged within 23 h after surgery and few patients developed a major complication. In addition to the clinical benefits of the program, patient satisfaction was high as the vast majority of patients appraised the program positively.

## Supplementary Information

Supplementary Information.

## Data Availability

Data supporting the results reported in the article can be accessed and electronically shared upon written request to the corresponding author either via email or post. Prior to data sharing, all data will be anonymized.

## References

[1] Gustafsson U (2019). Guidelines for Perioperative Care in Elective Colorectal Surgery: Enhanced Recovery After Surgery (ERAS®) Society Recommendations: 2018. World J Surg.

[2] Spanjersberg, W. R., Reurings, J., Keus, F. & van Laarhoven, C. J. Fast track surgery versus conventional recovery strategies for colorectal surgery. *Cochrane Database Syst. Rev.*, CD007635 (2011). 10.1002/14651858.CD007635.pub2 PMC13061361 21328298

[3] Delaney CP (2001). 'Fast track' postoperative management protocol for patients with high co-morbidity undergoing complex abdominal and pelvic colorectal surgery. Br. J. Surg..

[4] Basse L, Thorbøl JE, Løssl K, Kehlet H (2004). Colonic surgery with accelerated rehabilitation or conventional care. Dis. Colon Rectum.

[5] Thiele, R. H., MD *et al*. Standardization of Care: Impact of an Enhanced Recovery Protocol on Length of Stay, Complications, and Direct Costs after Colorectal Surgery. *Journal of the American College of Surgeons* **220**, 430–443 (2015). 10.1016/j.jamcollsurg.2014.12.042 25797725

[6] Dekker, J. W. Indicatorenset. DCRA verslagjaar 2020. *Dutch Institute for Clinical Auditing* (2020).

[7] Bokey EL (1995). Postoperative morbidity and mortality following resection of the colon and rectum for cancer. Dis. Colon Rectum.

[8] Haverkamp M, de Roos M, Ong K (2012). The ERAS protocol reduces the length of stay after laparoscopic colectomies. Surg. Endosc..

[9] Levy B, Scott M, Fawcett W, Rockall T (2009). 23-hour-stay laparoscopic colectomy. Dis. Colon Rectum.

[10] Rossi G (2013). Two-day hospital stay after laparoscopic colorectal surgery under an enhanced recovery after surgery (ERAS) pathway. World J. Surg..

[11] Gignoux B (2019). Short-term outcomes of ambulatory colectomy for 157 consecutive patients. Ann. Surg..

[12] Lee, L. *et al*. Enhanced recovery 2.0—same day discharge with mobile app follow-up after minimally invasive colorectal surgery. *Ann. Surg.* **Publish Ahead of Print** (2021). 10.1097/SLA.0000000000004962 34091514

[13] Saadat LV (2020). Twenty-three-hour-stay colectomy without increased readmissions: An analysis of 1905 cases from the national surgical quality improvement program. World J. Surg..

[14] Dobradin A, Ganji M, Alam SE, Kar PM (2013). Laparoscopic colon resections with discharge less than 24 hours. J. Soc. Laparoendosc. Surg..

[15] Lawrence, J. K., MD *et al*. Discharge within 24 to 72 hours of colorectal surgery is associated with low readmission rates when using enhanced recovery pathways. *J. Am. Coll. Surg.* **216**, 390–394 (2013). 10.1016/j.jamcollsurg.2012.12.014 23352608

[16] Emmanuel A, Chohda E, Botfield C, Ellul J (2018). Accelerated discharge within 72 hours of colorectal cancer resection using simple discharge criteria. Ann. R. Coll. Surg. Engl..

[17] World Medical Association Declaration of Helsinki (2013). Ethical principles for medical research involving human subjects. JAMA J. Am. Med. Assoc..

[18] Kruizenga, H. M., Seidell, J. C., de Vet, H. C. W., Wierdsma, N. J. & van Bokhorst–de van der Schueren, M.A.E. Development and validation of a hospital screening tool for malnutrition: the short nutritional assessment questionnaire (SNAQ©). *Clin. Nutr. (Edinburgh, Scotland)* **24**, 75–82 (2005). 10.1016/j.clnu.2004.07.015 15681104

[19] Parker SG (2018). What is comprehensive geriatric assessment (CGA)? An umbrella review. Age Ageing.

[20] de Azevedo, José Gonçalves Moreira *et al*. Laparoscopic colorectal surgery and discharge within 24 h—Who is at risk for readmission? *Colorectal Dis.* **23**, 2714–2722 (2021). 10.1111/codi.15791 34174142

[21] Faiz O (2011). Hospital stay amongst patients undergoing major elective colorectal surgery: predicting prolonged stay and readmissions in NHS hospitals. Colorectal Dis..

[22] Gustafsson, U. O. *et al*. Adherence to the enhanced recovery after surgery protocol and outcomes after colorectal cancer surgery. *Arch. Surg. (Chicago. 1960)* **146**, 571–577 (2011). 10.1001/archsurg.2010.309 21242424

[23] Kim MK (2019). Comparison of the effects of an ERAS program and a single-port laparoscopic surgery on postoperative outcomes of colon cancer patients. Sci. Rep..

[24] Henneman D (2013). hospital variation in failure to rescue after colorectal cancer surgery: Results of the dutch surgical colorectal audit. Ann. Surg. Oncol..

[25] Tweed TTT (2021). Reducing hospital stay for colorectal surgery in ERAS setting by means of perioperative patient education of expected day of discharge. Int. J. Colorectal Dis..

[26] Bednarski BK (2019). Randomized clinical trial of accelerated enhanced recovery after minimally invasive colorectal cancer surgery (RecoverMI trial). Br. J. Surg..

[27] van Brunschot DMD (2016). What is the evidence for the use of low-pressure pneumoperitoneum? A systematic review. Surg. Endosc..

[28] Hua, J., Gong, J., Yao, L., Zhou, B., & Song, Z. Low-pressure versus standard-pressure pneumoperitoneum for laparoscopic cholecystectomy: A systematic review and meta-analysis. *Am. J. Surg.* **208**, 143–150 (2014). 10.1016/j.amjsurg.2013.09.027 24503370

[29] Singla, S., Mittal, G., Raghav & Mittal, R. K. Pain management after laparoscopic cholecystectomy-a randomized prospective trial of low pressure and standard pressure pneumoperitoneum. *J. Clin. Diagn. Res.* **8**, 92–94 (2014). 10.7860/JCDR/2014/7782.4017 PMC3972609 24701492

[30] Radosa J (2019). Impact of different intraoperative CO2 pressure levels (8 and 15 mmHg) during laparoscopic hysterectomy performed due to benign uterine pathologies on postoperative pain and arterial pCO2: A prospective randomised controlled clinical trial. BJOG.

[31] Hanna MH (2015). Laparoscopic right hemicolectomy: Short- and long-term outcomes of intracorporeal versus extracorporeal anastomosis. Surg. Endosc..

[32] Ricci C (2016). A critical and comprehensive systematic review and meta-analysis of studies comparing intracorporeal and extracorporeal anastomosis in laparoscopic right hemicolectomy. Langenbecks Arch. Surg..

[33] Trépanier M (2019). Intracorporeal versus extracorporeal anastomosis for right colectomy does not affect gastrointestinal recovery within an enhanced recovery after surgery program. Surg. Endosc..

[34] Zheng J (2021). Comparison of intracorporeal and extracorporeal anastomosis and resection in right colectomy: A systematic review and meta-analysis. Langenbecks Arch. Surg..

[35] Liao C (2021). Short- and medium-term outcomes of intracorporeal versus extracorporeal anastomosis in laparoscopic right colectomy: A propensity score-matched study. World J. Surg. Oncol..

[36] Frasson M (2015). Risk factors for anastomotic leak after colon resection for cancer: Multivariate analysis and nomogram from a multicentric, prospective, national study with 3193 patients. Ann. Surg..

[37] Kim, J. S., Cho, S. Y., Min, B. S. & Kim, N. K. Risk factors for anastomotic leakage after laparoscopic intracorporeal colorectal anastomosis with a double stapling technique. *J. Am. Coll. Surg.* **209**, 694–701 (2009). 10.1016/j.jamcollsurg.2009.09.021 19959036

[38] Orcutt ST (2012). Use of a Pfannenstiel incision in minimally invasive colorectal cancer surgery is associated with a lower risk of wound complications. Tech. Coloproctol..

