# Author Correction: Accelerated 23-h enhanced recovery protocol for colon surgery: the CHASE-study

**DOI:** 10.1038/s41598-023-32196-1

**Published:** 2023-04-04

**Authors:** Thaís T. T. Tweed, Misha A. T. Sier, Imane Daher, Maikel J. A. M. Bakens, Johan Nel, Nicole D. Bouvy, James van Bastelaar, Jan H. M. B. Stoot

**Affiliations:** 1 Department of Gastrointestinal Surgery, Zuyderland Medical Center, Dr. H. Van Der Hoffplein 1, 6162 BG Sittard-Geleen, The Netherlands; 2 Department of Surgery, Maastricht University Medical Center, P. Debeyelaan 25, 6229 HX Maastricht, The Netherlands

Correction to: *Scientific Reports* https://doi.org/10.1038/s41598-022-25022-7, published online 01 December 2022

The original version of this Article contained an error, where “(CD ≥ IIIb)” was incorrectly given as “(CD > IIIb)” in the Results section.

As a result, in the Result, under the subheading ‘Surgical outcomes’,

“Serious adverse events (CD > IIIb) occurred in two patients (4.9%) in the CHASE cohort requiring reoperation;”

now reads:

“Serious adverse events (CD ≥ IIIb) occurred in two patients (4.9%) in the CHASE cohort requiring reoperation;”

and,

“In comparison to the retrospective cohort, no difference was observed in complication rate (*p * value = 0.565), but severe complications (CD > IIIb) were observed more frequently in the retrospective cohort.”

now reads:

“In comparison to the retrospective cohort, no difference was observed in complication rate (*p * value = 0.565), but severe complications (CD ≥ IIIb) were observed more frequently in the retrospective cohort.”

The original Article has been corrected.

