# Supplementary material for: Accelerated 23-h enhanced recovery protocol for colon surgery: the CHASE-study
Source: Sci Rep. 2022 Dec 1;12:20707. doi: 10.1038/s41598-022-25022-7 (PMC9715541; doi:10.1038/s41598-022-25022-7)
Supplement: Supplementary file 1 — Supplementary Information. [file 41598_2022_25022_MOESM1_ESM.docx]

**Supplementary Information**

**Supplementary form S1.1 CHASE evaluation form**

Dear sir/madam,

We would like to know how you experienced the 23-hour accelerated recovery program. We ask you to fill in this evaluation form. With your information we can evaluate and improve the quality of this program. You still have the opportunity to share comments, complaints and ideas that you have not been able to share before.

1. **What did you think of the information provision regarding the 23-hour accelerated recovery program?** (on a scale from 1 to 10; 1 = very bad; 10 = very good)

| 1 | 2 | 3 | 4 | 5 | 6 | 7 | 8 | 9 | 10 |
| --- | --- | --- | --- | --- | --- | --- | --- | --- | --- |
| 0 | 0 | 0 | 0 | 0 | 0 | 0 | 0 | 0 | 0 |

1. **How did you experience the care during hospital stay, e.g. contact with nurses and doctors?** (on a scale from 1 to 10; 1 = very bad; 10 = very good)

| 1 | 2 | 3 | 4 | 5 | 6 | 7 | 8 | 9 | 10 |
| --- | --- | --- | --- | --- | --- | --- | --- | --- | --- |
| 0 | 0 | 0 | 0 | 0 | 0 | 0 | 0 | 0 | 0 |

1. **Was the aftercare after discharge arranged as desired?** (on a scale from 1 to 10; 1 = very bad; 10 = very good)

| 1 | 2 | 3 | 4 | 5 | 6 | 7 | 8 | 9 | 10 |
| --- | --- | --- | --- | --- | --- | --- | --- | --- | --- |
| 0 | 0 | 0 | 0 | 0 | 0 | 0 | 0 | 0 | 0 |

1. **Did you have to contact the hospital because your health deteriorated after surgery? If yes, how often?**

| 1 | 2 | 3 | 4 | 5 | 6 | 7 | 8 | 9 | 10 |
| --- | --- | --- | --- | --- | --- | --- | --- | --- | --- |
| 0 | 0 | 0 | 0 | 0 | 0 | 0 | 0 | 0 | 0 |

1. **How do you rate the entire 23-hour accelerated recovery program?** (on a scale from 1 to 10; 1 = very bad; 10 = very good)

| 1 | 2 | 3 | 4 | 5 | 6 | 7 | 8 | 9 | 10 |
| --- | --- | --- | --- | --- | --- | --- | --- | --- | --- |
| 0 | 0 | 0 | 0 | 0 | 0 | 0 | 0 | 0 | 0 |

Comments:

**Supplementary Table S1.2 Reasons for prolonged admission (> 23 hours)**

| Reason for prolonged admission | Number of patients (%) |
| --- | --- |
| Rectal blood loss | 3 (7.3%) |
| Bladder retention | 2 (4.9%) |
| Pain | 2 (4.9%) |
| Nausea | 1 (2.4%) |

**Supplementary Table S1.3 Complications specifications**

| Complication | CHASE cohort, n (%)  N= 13 | Retrospective cohort, n (%)  N=20 |
| --- | --- | --- |
| Anastomotic leakage | 1 (7.7%) | 4 (20.0%) |
| Bowel ischemia | 0 | 1 (5.0%) |
| Trocar hernia | 1 (7.7%) | 0 |
| Abscess drainage | 0 | 2 (10%) |
| Rectal blood loss | 4 (30.8%) | 0 |
| Anemia | 0 | 2 (10%) |
| Bladder retention | 4 (30.8%) | 1 (5.0%) |
| Ileus | 2 (15.4%) | 1 (5.0%) |
| Infection  Urinary tract infection  Pneumonia  Pelvic inflammatory disease  Infection e.c.i. | 1 (7.7%)  1 (7.7%)  0  0 | 0  4 (20.0%)  1 (5.0%)  1 (5.0%) |
| Atrial fibrillation  Tachycardia  Embolus | 0  0  0 | 2 (10%)  1 (5.0%)  1 (5.0%) |
| Electrolyte deficiencies | 1 (7.7%) | 2 (10.0%) |

**Supplementary Table S1.4. Specification of readmission & Types of resections.**

| **Reason readmission**  **Total readmissions** | **CHASE cohort, *n* (%)**  ***n* = 7 (17.1%)** | **Retrospective cohort, *n* (%)**  ***n* = 4 (5.3%)** | |
| --- | --- | --- | --- |
| Anastomotic leakage | 1 (2.4%) | | 1 (1.3%) |
| Trocar hernia | 1 (2.4%) | | 0 |
| Rectal blood loss | 2 (4.9%) | | 0 |
| Abscess | 0 | | 1 (1.3%) |
| Ileus | 1 (2.4%) | | 1 (1.3%) |
| Biliary pancreatitis | 0 | | 1 (1.3%) |
| Pneumonia | 1 (2.4%) | | 0 |
| Dizziness | 1 (2.4%) | | 0 |
| **Types of resection** | **CHASE cohort, *n* (%)** | | **Retrospective cohort, *n* (%)** |
| Left hemicolectomy | 1 (2.4%) | | 2 (2.7%) |
| Right hemicolectomy | 2 (4.9%) | | 1 (1.3%) |
| High anterior resection | 3 (7.3%) | | 1 (1.3%) |
| Transverse colectomy | 1 (2.4%) | | 0 |
